# Comparative Assessment of Economic Burden of Disease in Relation to Out of Pocket Expenditure

**DOI:** 10.3389/fpubh.2019.00009

**Published:** 2019-01-29

**Authors:** Shivendra Sangar, Varun Dutt, Ramna Thakur

**Affiliations:** School of Humanities and Social Sciences, Indian Institute of Technology Mandi, Mandi, India

**Keywords:** economic burden, impoverishment, ailments, infections, morbidity

## Abstract

**Background:** The economic costs associated with morbidity pose a great financial risk on the population. Household's over-dependence on out-of-pocket (OOP) health expenditure and their inability to cope up with the economic costs associated with illness often push them into poverty. The current paper aims to measure the economic burden and resultant impoverishment associated with OOP health expenditure for a diverse set of ailments in India.

**Methods:** Cross-sectional data from National Sample Survey Organization (NSSO) 71st Round on “Key Indicators of Social Consumption: Health” has been employed in the study. Indices, namely the payment headcount, payment gap, concentration index, poverty headcount and poverty gap, are defined and computed. The measurement of catastrophic burden of OOP health expenditure is done at 10% threshold level.

**Results:** Results of the study reveal that collectively non-communicable diseases (NCDs) have higher economic and catastrophic burden, individually infections rather than NCDs such as Cardio Vascular Diseases and cancers have a higher catastrophic burden and resultant impoverishment in India. Ailments such as gastro-intestinal, respiratory, musco-skeletal, obstetrics, and injuries also have a substantial economic burden on population and push them below the poverty line. Results also show that despite the pro-poor concentration of infections, their economic burden is more concentrated among the wealthier consumption groups.

**Conclusion:** The study concludes that universal health coverage through adequate provision of pooled resources for health care and community-based health insurance is critical to reduce the economic burden and impoverishment related to OOP health expenditure. Measures should also be instituted to insulate people from economic burden on morbidity, especially the NCDs.

## Background

The economic costs associated with morbidity pose a great financial risk on population (1). Direct spending on health care discourages people from using health care services and encourages them to postpone their health care needs (2, 3). In the majority of LMICs, limited resources for health care and lack of protection against catastrophic health spending have led to the over-reliance on OOP health expenditure (4).

Household's overly dependence on out-of-pocket (OOP) health expenditure and their inability to cope up with the economic costs of illness often push them into poverty (5, 6). Households faced with this situation face enormous financial liability and are devoid of adequate means for other essential needs such as food and education (7, 8). Further, rural dwelling, low socioeconomic status and outpatient care also contribute to the increasing economic burden of illness (9, 10).

There are few studies which have talked about this issue by taking into consideration either NCDs or infectious diseases without analyzing its impact on impoverishment (6, 11, 12). There is no study which has analyzed the monetary burden and impoverishment impact of different sets of NCDs and infectious diseases separately. Therefore, this study aims at filling this gap in the literature by analyzing the economic burden and resultant impoverishment due to OOP health expenditure for a diverse set of ailments by employing the recent health expenditure survey in India.

## Materials and Methods

### Data

The current study employed a nationally representative data from National Sample Survey Organization (NSSO), 71st Round (2004) on Key indicators of social consumption: Health (13). The survey comprises 65,932 sample households consisting a population of 0.33 million persons. The survey adopted a stratified multistage sample design, using census villages for the rural areas and urban blocks for the urban areas as the first-stage units (FSUs), and households as the second-stage units. The reference period for inpatient and outpatient care is 365 and 15 days, respectively. For the analysis, the OOP health expenditure for inpatient and outpatient care is converted into monthly figures and added together to get the total OOP health expenditure. In this study, OOP health expenditure is calculated by deducting the amount of reimbursement from total health expenditure. Respective sample weights have been applied in the calculation of the results. For the analysis, we have taken 16 groups of different ailments (13). The analysis is done on Stata 14 (14).

### Methods

#### Measuring the Economic Burden of Morbidity

The economic burden of morbidity has been calculated in terms of headcount and payment gap (15). Headcount (HC) measures the percentage of population incurring OOP health expenditure. It is measured by $HC=\;\frac{1}{N}\overset{N}{\underset{i=1}{\sum }}n_{i}$, where N is the sample size, HC is the headcount and n is number of persons incurring OOP health expenditure. The payment gap is explained as the share of OOP health expenditure in total consumption expenditure is given by $G=\;\frac{H}{TCE}\;*100$, S is the payment gap, H is the OOP health expenditure, TCE is the total consumption expenditure. Measurement of catastrophic burden of OOP health expenditure (headcount and payment gap) is done at 10% threshold level of TCE which has been a standard benchmark in the literature (15, 16). Catastrophic headcount is a fraction of the population whose OOP health expenditure as a proportion of TCE exceed the given threshold. Whereas, catastrophic payment gap measures the average degree by which OOP health expenditure as a proportion of TCE exceeds the threshold level. The concentration index has been used to determine whether the poor incur more OOP health expenditure or the rich (17). Concentration index C_E_ and C_O_ (for headcount and payment gap, respectively) as given by the following formula: *CI* = (*p*_1_*L*_2_− *p*_2_*L*_1_) + (*p*_2_*L*_3_− *p*_3_*L*_2_)+…+(*p*_*t*−1_*L*_*t*_− *p*_*t*_*L*_*t*−1_), Where CI is the concentration index, p_t_ is the cumulative percentage of the population ranked by monthly consumption expenditure in group t, L_t_ is the corresponding health variable.

#### Measuring the Impoverishment Impact of Morbidity

Poverty headcount impact measures the fraction of population falling below the poverty line due to OOP health expenditure. The poverty impact in terms of headcount is measured as *PI*^*HC*^ = *HC*^*Post*^− *HC*^*Pre*^, where PI^HC^ is the poverty headcount impact, Z^Pre^ be the pre-payment poverty line. Then P^Pre^ = 1 if x < Z^Pre^ $HC^{Pre\;}=\;\frac{1}{N}\overset{N}{\underset{i=1}{\sum }}P^{Pre}$, P^Pre^ is the pre-payment poverty headcount, HC^Post^ and HC^Post^ are the post and pre-payment poverty headcount. Poverty impact in terms of gap measures the average shortfall due to OOP health expenditure from the existing poverty line. It is given as *PI*^*G*^ = *G*^*Post*^−*G*^*Pre*^, where PI^G^ is the poverty gap impact, g^Pre^ is the pre-payment gap, that is equal to x—Z^Pre^ if x < Z^Pre^, and zero otherwise, $G^{Pre}=\;\frac{1}{N}\;\overset{N}{\underset{i=1}{\sum }}g^{Pre}\;$, G^Post^ and G^Pre^ are the post and pre-payment poverty gap.

## Results

### Economic Burden of Morbidity in India

Table 1 reports the economic and catastrophic burden of OOP health expenditure incurred on different ailments. Although, collectively NCDs have higher economic burden, in case of individual ailments a significant proportion of population reported OOP health expenditure in case of infections followed by respiratory, CVDs, musco-skeletal, gastro-intestinal, psychiatric, and injuries. Although in lesser proportions, OOP health expenditure is also reported in other categories of ailments. Similarly, the payment gap as share of OOP health expenditure in TCE is also higher among infections as compared to other ailments. Similar to the economic burden, the catastrophic burden reported at 10% threshold level is relatively higher in case of infections than other ailments. However, collectively NCDs have higher catastrophic impact on the population. In case of infections, the negative value of C.I_E_ and the positive value of C.I_O_ reveal that despite its pro-poor concentration, it is the wealthier consumption groups which spends more on the treatment. However, in case of NCDs, especially CVDs and cancer, the positive values of CI_E_ and CI_O_ reveal a pro-rich concentration of headcount as well as OOP health expenditure.

**Table 1:** Economic burden of morbidity in India.

**Type of ailments**	**Population reporting OOP (%)**	C.I_E_^*^	**Payment gap (%)**	C.I_O_^**^	**Catastrophic headcount 10%**	**Catastrophic payment gap 10%**
Infections	14.1 (13.5 to 14.7)	−0.034 (−0.055 to −0.014)	2.2 (2.1 to 2.4)	0.058 (0.039 to 0.076)	6.6 (6.2 to 7.0)	1.2 (1.0 to 1.3)
Cancers	0.4 (0.3 to 0.5)	0.282 (0.243 to 0.322)	0.4 (0.3 to 0.5)	0.441 (0.410 to 0.472)	0.3 (0.2 to 0.4)	0.3 (0.2 to 0.4)
CVDs	5.3 (5.0 to 5.7)	0.339 (0.224 to 0.452)	1.7 (1.5 to 1.9)	0.524 (0.455 to 0.593)	2.8 (2.5 to 3.0)	1.0 (0.9 to 1.1)
Injuries	2.3 (2.1 to 2.4)	0.124 (0.081 to 0.166)	0.8 (0.7 to 0.9)	0.293 (0.260 to 0.325)	1.3 (1.2 to 1.4)	0.6 (0.5 to 0.7)
Respiratory	6.0 (5.6 to 6.5)	0.027 ( to 0.010 to 0.065)	1.0 (0.8 to 1.3)	0.130 (0.100 to 0.159)	2.4 (2.1 to 2.7)	0.6 (0.4 to 0.7)
Gastro–intestinal	4.2 (4.0 to 4.5)	0.055 (0.014 to 0.095)	1.0 (0.8 to 1.1)	0.201 (0.173 to 0.229)	2.3 (2.1 to 2.6)	0.7 (0.5 to 0.8)
Blood Disorders	0.7 (0.5 to 0.8)	0.113 (−0.014 to 0.239)	0.2 (0.1 to 0.3)	0.239 (0.166 to 0.311)	0.4 (0.3 to 0.5)	0.1 (0.08 to 0.2)
Endocrine	0.7 (0.5 to 0.8)	0.113 (−0.009 to 0.233)	0.2 (0.1 to 0.3)	0.239 (0.166 to 0.312)	0.4 (0.3 to 0.5)	0.1 (0.08 to 0.2)
Psychiatric	2.7 (2.4 to 3.0)	0.075 (0.026 to 0.124)	0.8 (0.7 to 0.9)	0.267 (0.230 to 0.303)	1.4 (1.3 to 1.6)	0.5 (0.4 to 0.6)
Eye	1.1 (1.0 to 1.3)	0.088 (0.015 to 0.159)	0.2 (0.1 to 0.3)	0.201 (0.147 to 0.256)	0.5 (0.4 to 0.6)	0.1 (0.06 to 0.02)
Ear	0.3 (0.2 to 0.4)	0.167 (0.035 to 0.298)	0.1 (0.02 to 1.7)	0.237 (0.125 to 0.349)	0.2 (0.1 to 0.3)	0.1 (0.01 to 1.9)
Skin	1.1 (1.0 to 1.2)	0.028 (−0.060 to 0.116)	0.2 (0.1 to .0.3)	0.124 (0.058 to 0.190)	0.5 (0.4 to 0.6)	0.1 (0.08 to 1.1)
Musco–skeletal	4.6 (4.3 to 5.0)	0.010 (0.005 to 0.016)	1.0 (0.09 to 1.1)	0.265 (0.230 to 0.299)	2.4 (2.1 to 2.7)	0.6 (0.5 to 0.7)
Genito–urinary	1.6 (1.4 to 1.8)	0.214 (0.153 to 0.274)	0.7 (0.6 to 0.8)	0.293 (0.256 to 0.332)	1.1 (1.0 to 1.2)	0.4 (0.3 to 0.5)
Obstetrics	1.1 (1.0 to 1.2)	−0.003 (−0.004 to −0.002)	0.2 (0.1 to 0.3)	0.120 (0.069 to 0.171)	0.5 (0.4 to 0.6)	0.1 (0.04 to 1.9)
Others	1.1 (1.0 to 1.3)	0.195 (0.118 to 0.0.271)	0.3 (0.2 to 0.4)	0.352 (0.292 to 0.412)	0.5 (0.4 to 0.6)	0.2 (0.1 to 0.3)
Total	47.3 (46.4 to 48.4)	0.064 (0.053 to 0.075)	11.0 (10.5 to 11.5)	0.251 (0.239 to 0.262)	23.7 (22.9 to 24.4)	7.8 (7.3 to 8.2)

*The figures are based on author's calculations from NSSO 71st Round. Values in parentheses are 95% confidence interval*.

* *C.I_E_ is the concentration index for headcount*.

** *C.I_O_ is the concentration index for payment gap*.

### Poverty Impact of Morbidity in India

Table 2 presents the poverty impact of OOP health expenditure incurred on different ailments in terms of headcount and payment gap. Poverty impact in terms of headcount is highest in case of infections, followed by CVDs and gastro-intestinal, musco-skeletal, respiratory, and injuries. Similarly, the poverty impact in terms of payment gap is also significantly higher in infections. It shows that the average shortfall from the poverty line is higher in case of infections than other ailments. Ailments consisting of gastro, musco-skeletal, respiratory, CVDs, and injuries also have higher poverty gap impact. Some other ailments such as skin, blood disorders, eye and ear also marginally contribute toward impoverishment.

**Table 2:** Poverty impact of morbidity in India.

**Type of ailments**	**Poverty impact headcount (%)**	**Poverty impact (Number)**	**Poverty impact gap (INR)**
Infections	2.1 (1.9–2.3)	23,543,134	14.9 (0.18) (13.6–16.3)
Cancers	0.2 (0.1–0.3)	2,242,203	1.2 (0.01) (0.8–1.5)
CVDs	0.8 (0.7–0.9)	8,968,813	5.0 (0.06) (4.4–5.6)
Injuries	0.5 (0.4–0.7)	5,605,508	3.9 (0.05) (3.4–4.5)
Respiratory	0.6 (0.5–0.8)	6,726,610	5.5 (0.07) (4.1–6.9)
Gastro–intestinal	0.8 (0.7–0.9)	8,968,813	5.9 (0.07) (5.0–6.9)
Blood Disorders	0.1 (0.07–1.3)	1,121,102	1.0 (0.01) (0.6–1.3)
Endocrine	0.5 (0.4–0.6)	5,605,508	2.6 (0.03) (2.1–3.1)
Psychiatric	0.5 (0.4–0.6)	5,605,508	3.8 (0.05) (3.3–4.3)
Eye	0.2 (0.09–0.3)	2,242,203	0.8 (0.01) (0.5–1.1)
Ear	0.1 (0.02–0.07)	1,121,102	0.3 (0.01) (0.1–0.5)
Skin	0.1 (0.01–0.3)	1,121,102	1.0 (0.01) (0.8–1.3)
Musco–skeletal	0.7 (0.6–0.9)	7,847,711	5.6 (0.07) (4.5–7.0)
Genito	0.4 (0.3–0.5)	4,484,406	2.8 (0.03) (2.1–3.4)
Obstetrics	0.2 (0.1–0.3)	2,242,203	1.1 (0.01) (0.7–1.5)
Others	0.2 (0.1–0.3)	2,242,203	1.3 (0.02) (0.9–1.7)
Total	8.0 (7.6–8.4)	89,688,129	56.7 (0.70) (53.8–59.9)

*The figures are based on author's calculations from NSSO 71st Round. Values in parentheses are 95% confidence interval. INR, Indian National Rupee. INR has been converted into Euro for the year 2014*.

## Discussion

Overall, the results of the study reveal that NCDs such as CVDs, cancers, etc. have the higher catastrophic burden and resultant impoverishment in India. Although individually CVDs have a significant economic burden and high poverty impact, it is less than infections. Ailments such as gastro-intestinal, respiratory, musco-skeletal, obstetrics and injuries also have a substantial economic burden on population and push them below the poverty line. Infections have higher poverty impact because the population affected with the same is more concentrated around the poverty line. A smaller increase in OOP health expenditure pushes the larger proportion of population below the poverty line.

Although it is true that the burden of NCDs is increasing in India and cumulatively they have higher catastrophic burden but it is the infectious diseases which push more people into the quagmire of poverty (18). Studies from other LMICs reveal that the economic burden and resultant impoverishment due to OOP health expenditure has been relatively high in case of NCDs such as CVDs, cancer, diabetes and stroke (19, 20). Further, many countries in Africa have higher incidence of catastrophic health expenditure due to infectious disease like Malaria and Tuberculosis (21, 22). In LMICs, lack of access to health services, poor quality of care and high user charges contribute to higher OOP health expenditure (12). Further, inadequate public spending on health care and poor implementation of publicly financed health insurance schemes (PFHIs) have further accentuated the problem of health care financing in India (23, 24).

The high catastrophic burden and resultant impoverishment associated with morbidity highlight the need for a better financial protection mechanism in India, particularly for the poor and vulnerable. Universal health coverage (UHC) is regarded as a critical path for improving the health outcomes and providing financial protection against the catastrophic health expenditure (25). It is a comprehensive health system approach that helps to provide improved access to health care services which significantly improves the health outcomes (26). UHC can be achieved through a matured health system that can provide sufficient and continuous pooled resources for health (27). Apart from improving access to health care services, policy makers must focus on extending quality care, especially to poor families (28). LMICs can implement community-based health insurance (CBHI) which can go a long way in achieving UHC (29).

The future plan of the research will be to measure the economic burden of ailments at different threshold levels. Along with-it different sources of finance used to cope up with OOP health expenditure for different ailments will also be studied. A comparative analysis with previous data rounds may also be useful.

## Data Availability Statement

This paper is based on anonymized survey data collected by the National Sample Survey organization (NSSO), a department of the Ministry of Statistics and Programme Implementation, Government of India. Data is available in the public domain. The data is already available in publicly available repositories to individuals both at national and international level through http://www.mospi.gov.in/

## Author Contributions

RT and SS conceived the idea with inputs from VD. SS performed the statistical analysis and prepared the initial draft of the manuscript. RT and VD assisted in the revision of the manuscript. All authors read and approved the final manuscript.

### Conflict of Interest Statement

The authors declare that the research was conducted in the absence of any commercial or financial relationships that could be construed as a potential conflict of interest.
